# CD39 abrogates platelet-derived factors induced IL-1β expression in the human placenta

**DOI:** 10.3389/fcell.2023.1183793

**Published:** 2023-05-30

**Authors:** Désirée Forstner, Jacqueline Guettler, Beatrice A. Brugger, Freya Lyssy, Lena Neuper, Christine Daxboeck, Gerhard Cvirn, Julia Fuchs, Kristin Kraeker, Alina Frolova, Daniela S. Valdes, Christina Stern, Birgit Hirschmugl, Herbert Fluhr, Christian Wadsack, Berthold Huppertz, Olivia Nonn, Florian Herse, Martin Gauster

**Affiliations:** ^1^ Division of Cell Biology, Histology and Embryology, Gottfried Schatz Research Center, Medical University of Graz, Graz, Austria; ^2^ Division of Medicinal Chemistry, Otto Loewi Research Center, Medical University of Graz, Graz, Austria; ^3^ Division of Medical Physics and Biophysics, Gottfried Schatz Research Center, Medical University of Graz, Graz, Austria; ^4^ Experimental and Clinical Research Center, A Cooperation Between the Max‐Delbrück‐Center for Molecular Medicine in the Helmholtz Association and the Charité—Universitätsmedizin Berlin, Berlin, Germany; ^5^ Charité – Universitätsmedizin Berlin, Corporate Member of Freie Universität Berlin and Humboldt‐Universität zu Berlin, Berlin, Germany; ^6^ Max‐Delbrück‐Center for Molecular Medicine in the Helmholtz Association (MDC), Berlin, Germany; ^7^ Institute of Molecular Biology and Genetic of NASU, Kyiv, Ukraine; ^8^ Department of Obstetrics and Gynecology, Medical University of Graz, Graz, Austria

**Keywords:** ectonucleotidases, placenta, platelet-derived factors, adenosine triphosphate (ATP), preecclampsia

## Abstract

Tissue insults in response to inflammation, hypoxia and ischemia are accompanied by the release of ATP into the extracellular space. There, ATP modulates several pathological processes, including chemotaxis, inflammasome induction and platelet activation. ATP hydrolysis is significantly enhanced in human pregnancy, suggesting that increased conversion of extracellular ATP is an important anti-inflammatory process in preventing exaggerated inflammation, platelet activation and hemostasis in gestation. Extracellular ATP is converted into AMP, and subsequently into adenosine by the two major nucleotide-metabolizing enzymes CD39 and CD73. Here, we aimed to elucidate developmental changes of placental CD39 and CD73 over gestation, compared their expression in placental tissue from patients with preeclampsia and healthy controls, and analyzed their regulation in response to platelet-derived factors and different oxygen conditions in placental explants as well as the trophoblast cell line BeWo. Linear regression analysis showed a significant increase in placental CD39 expression, while at the same time CD73 levels declined at term of pregnancy. Neither maternal smoking during first trimester, fetal sex, maternal age, nor maternal BMI revealed any effects on placental CD39 and CD73 expression. Immunohistochemistry detected both, CD39 and CD73, predominantly in the syncytiotrophoblast layer. Placental CD39 and CD73 expression were significantly increased in pregnancies complicated with preeclampsia, when compared to controls. Cultivation of placental explants under different oxygen conditions had no effect on the ectonucleotidases, whereas presence of platelet releasate from pregnant women led to deregulated CD39 expression. Overexpression of recombinant human CD39 in BeWo cells decreased extracellular ATP levels after culture in presence of platelet-derived factors. Moreover, platelet-derived factors-induced upregulation of the pro-inflammatory cytokine, interleukin-1β, was abolished by CD39 overexpression. Our study shows that placental CD39 is upregulated in preeclampsia, suggesting an increasing demand for extracellular ATP hydrolysis at the utero-placental interface. Increased placental CD39 in response to platelet-derived factors may lead to enhanced conversion of extracellular ATP levels, which in turn could represent an important anti-coagulant defense mechanism of the placenta.

## Introduction

Tissue insults in response to inflammation, hypoxia or ischemia, are accompanied by the release of purinergic mediators into the extracellular space, where they are involved in the regulation of immune cell functions, such as intercellular interactions, cytokine and chemokine secretion, surface antigen shedding, intracellular pathogen removal, and production of reactive oxygen species (Antonioli et al., 2013). Amongst released purinergic mediators, extracellular ATP serves as an important signaling molecule that modulates several pathological processes in scenarios of thrombosis and inflammation, such as chemotaxis, inflammasome induction and platelet activation (Allard et al., 2017). Extracellular ATP concentrations range between 400 and 700 nmol/L in healthy conditions, but can increase 3-fold in diseases such as preeclampsia (PE). PE is a multisystem pregnancy complication, characterized by new-onset hypertension (>140 mmHg systolic and/or >90 mmHg diastolic) in combination with proteinuria or other organ dysfunctions in the second half of pregnancy (Spaans et al., 2014; Brown et al., 2018). Extracellular ATP is converted into AMP, and subsequently into adenosine by the two major extracellular nucleotides-metabolizing enzymes CD39 (ectonucleoside triphosphate diphosphohydrolase-1, encoded by *ENTPD1*) and CD73 (ecto-5′-nucleotidase, encoded by *NT5E*). While the first step of extracellular ATP clearance is catalyzed by CD39, the second step of the ATP to adenosine conversion is catalyzed by CD73, which dephosphorylates extracellular AMP to adenosine (Allard et al., 2017). The expression and activity of both ectonucleotidases seems to be dynamically adapted to the (patho-) physiological environment, and it is increasingly recognized that altering this machinery can affect the outcome of several pathophysiological conditions, like atherosclerosis, autoimmune diseases, cancer, infections, and ischemia–reperfusion injury (Antonioli et al., 2013). Moreover, alterations in the expression and activity of these ectonucleotidases can contribute to disorders of thromboregulation, since the CD39/CD73 axis regulates hemostasis by converting a local pro-thrombotic ATP/ADP-rich environment into an anti-thrombotic adenosine-rich environment (Koszalka et al., 2004; Atkinson et al., 2006).

Indeed, pregnancy seems to be a condition extremely susceptible to ATP-induced inflammation, as shown in an ATP-infused rat model. According to that, ATP infusion induces a pro-inflammatory response leading to glomerular albuminuria exclusively in pregnant but not in non-pregnant control rats (Faas et al., 2010). In the human placenta, both CD39 and CD73 have been detected in the villous trophoblast compartment at term (Thomson et al., 1990; Jaakkola et al., 2000; Kittel et al., 2004; Zhu et al., 2018), and their role in the pathogenesis of preeclampsia seems currently rather controversial. While a study by Iriyama et al. did not show a difference in CD39 mRNA levels, but demonstrated significantly elevated mRNA, protein level and enzyme activity of CD73 in placentas from preeclampsia patients at term (Iriyama et al., 2015) patients at term, another recent study by Zhu et al. described significantly decreased placental CD39 expression in pregnancies complicated with late-onset preeclampsia (Zhu et al., 2018).

Deregulation of ectonucleotidases may lead to altered levels of extracellular ATP and adenosine. In healthy human pregnancy, maternal plasma adenosine, but not ATP levels are increased, probably as a result of increased platelet activation (Yoneyama et al., 2002a; Bakker et al., 2007). However, in preeclampsia, both maternal plasma ATP and adenosine levels are increased, 2.5-fold and 1.5-fold, respectively, resulting in a significantly increased ATP/adenosine ratio compared to controls (Spaans et al., 2014). Persistent high adenosine levels in preeclamptic patients are suggested to increase formation of nitric oxide and peroxynitrite anion, leading to endothelial dysfunction (Spaans et al., 2014). In this study, we aimed at elucidating the ectonucleotidases CD39 and CD73 regarding their placental expression during gestation, and the effects of oxygen and platelet-derived factors on their expression. Based on this data, we identified placental changes of both nucleotidases in cases suffering from preeclampsia.

## Methods

### Placental tissue collection

The study was approved by the ethical committee of the Medical University of Graz (26-132 ex 13/14 and 31-019 ex 18/19). Human first trimester placental tissues (Supplementary Table S3) were collected between week 5 and 12 of gestation with written informed consent from women undergoing legal elective surgical pregnancy terminations at a local gynecologist. Self-reported smoking status was verified by serum cotinine level measurements as recently described by Hoch et al. (2023). Pre-term and term placental tissues (Supplementary Table S3) were obtained with written informed consent mostly after caesarean section at the Department of Obstetrics and Gynecology at the University Hospital Graz. The control group comprised placentas delivered by caesarean section due to velamentous insertion of the cord, cervical insufficiency with no clinical evidence for infection or *placenta previa*. Placental villous tissue was rinsed in buffered saline and dissected into small pieces of approximately 5mm^3^, before the tissue pieces were snap frozen in liquid nitrogen and stored at −80°C until further processing or used for *ex vivo* cultures of placental villi. Baseline characteristics of the study population are shown in Supplementary Table S3.

### *Ex vivo* culture of placental villi

Placental villous tissue from human first and third trimester was processed within 1–4 h after the medical intervention. Tissue samples were thoroughly rinsed in buffered saline and dissected under the stereoscopic microscope into small pieces of approximately 1–2 mm (Forstner et al., 2020). Placental explants were transferred into 24-well dishes (Nunc) and cultured in 1 mL/well of DMEM/F12 (1:1, Gibco, life technologies; Paisley, United Kingdom) supplemented with 10% FCS (Gibco), penicillin/streptomycin (Gibco) and L-glutamine (Gibco) in a hypoxic workstation (BioSpherix Ltd.; Redfield, NY, United States) under indicated oxygen concentrations and 5% CO_2_ in a humidified atmosphere at 37°C for 24 h.

### Culture of trophoblast cell line BeWo

For *in vitro* studies, the trophoblast cell line BeWo, purchased from the European Collection of Cell Cultures (ECACC), was used. BeWo cells were cultured in DMEM/F12 (1:1, Gibco, life technologies; Paisley, United Kingdom) supplemented with 10% FCS (Gibco), 0.1 U/mL Penicillin and 0.1 μg/mL streptomycin (Gibco) and 1% (v/v) L-glutamine (Gibco; 20 mM 100X) in a humidified atmosphere of 5% CO_2_ at 37°C. Cells between passage 10 and 30 were used for *in vitro* experiments.

### Transfection of BeWo cells

For CD39 overexpression, BeWo cells with a density of 2 × 10^5^ cells per well were seeded in a 24-well dish (Nunc Lab-Tek, Thermo Fisher; NY, United State). After reaching about 80% confluency, cells were transfected using the K2^®^ Transfection System (Biontex Laboratories GmbH, Munich, Germany). Empty control plasmid and pRP-hENTPD1 (#VB220322-1057cfa, VectorBuilder) were diluted to a working concentration of 1 µg and pre-incubated with the K2^®^ transfection reagent (Biontex) for 15 min at room temperature (RT). To ensure an equal ratio between plasmid concentration and cell number in different experiments, the plasmid concentration was adapted to 0.75 µg or 0.075 µg for 1.5 × 10^5^ and 1.5 × 10^4^, respectively. Meanwhile, the culture medium was exchanged with serum-free DMEM/F12 (1:1, Gibco) supplemented with 1% L-glutamin (Gibco) and 1% penicillin/streptomycin (Gibco) and the plasmids were added for 24 h. Afterwards, cells were either lysed for analysis or further incubated with indicated treatments for 24 h.

### Isolation of human platelets and preparation of platelet releasate

Citrated whole blood samples (Vacuette^®^) from healthy pregnant donors before caesarean section was collected with written informed consent. Platelet counts were measured by means of the Sysmex KX-21 N Automated Hematology Analyzer (Sysmex, Illinois, IL, United State). In order to obtain platelet rich plasma (PRP), blood samples were centrifuged at 100 *g* for 15 min at RT. Afterwards, PRP was gently mixed with 13 mL platelet wash buffer consisting of aqua dest. With 128 mM NaCl (Supelco^®^, Merck; Darmstadt, Germany), 11 mM Glucose (Sigma) 7.5 mM Na_2_HPO_4_ (Merck), 4.8 mM sodium citrate (Sigma-Aldrich), 4.3 mM NaH_2_PO_4_ (Lactan; Graz, Austria), 2.4 mM citric acid (Merck) and 0.35% bovine serum albumin (Biowest; Nuaillé, France) with addition of 2.5 ng/μL prostaglandin (Cayman Chemical Company; Ann Arbor, MI, United State). After another centrifugation step at 3,000 rpm for 15 min at RT, the platelet pellet was resuspended in 7 mL platelet wash buffer and centrifuged again at 3,000 rpm for 15 min at RT. Platelet poor plasma (PPP) was removed and after resuspension of platelets in serum-free DMEM/F12 (1:1, Gibco) supplemented with penicillin/streptomycin (Gibco) and L-glutamine (Gibco) to the initial plasma volume, platelets were activated with 1 U/mL thrombin (Merck, Darmstadt KGaA, Germany) for 20 min. Afterwards, thrombin was inactivated with 1.1 U/mL hirudin (Merck, Darmstadt KGaA, Germany) and platelets were pelleted by centrifugation at 3,000 rpm for 15 min at RT. The supernatant, representing the so-called platelet releasate (PR), was collected and stored at −80°C until further usage. Efficiency of thrombin activation was confirmed by measuring released platelet factor 4 (PF4) and TGF-β and activity of PR was assessed by its ability to induce the well described TGF-β downstream target plasminogen activator inhibitor [PAI-1, encoded by *SERPINE1* (Dennler et al., 1998)] in BeWo cells (Supplementary Figure S1).

### Incubation of placental explants and trophoblast cells with platelet-derived factors

For incubation of explant cultures and trophoblast cells with platelet-derived factors, platelet releasate of 10 independent pregnant donors (mean gestational week 38.83 ± 2.59; mean platelet count 1.97 ± 4.78 × 10^5^ platelets/µL) was pooled and diluted 1:1 with culture medium. FBS to a final concentration of 10% was added to the platelet releasate-medium mixture. BeWo cells were transfected as described above and afterwards incubated in presence or absence of platelet releasate for 24 h. Placental explants were prepared as described above and treated with platelet releasate for 24 h. After incubation, cells as well as tissue lysates and supernatants were collected for further analysis.

### Isolation of placental primary cells

Primary trophoblasts were isolated from term placentas (n = 3) of healthy pregnancies as previously described by Loegl et al. (Loegl et al., 2017) and kindly provided by the Department of Obstetrics and Gynecology at the University Hospital Graz. In brief, placental villous tissue was minced and digested with trypsin/dispase/DNase (Gibco/Roche/Sigma) solution for 90 min. Afterwards, trophoblast cells were enriched by centrifugation at 4°C for 30 min at 300 g on a Percoll gradient (Sigma). Trophoblast cells were purified by immunodepletion of contaminating cells using beads conjugated to MCA-81 antibody (Serotec, Puchheim, Germany) against HLA-A, B and C. Trophoblast cells were then seeded in 6-well dishes (Thermo Fisher Scientific; Nunc) in 2 mL DMEM (Gibco) containing 10% FCS (Gibco) and 1% penicillin/streptomycin (Gibco) at 37°C and 5% CO_2_. Primary endothelial cells were isolated from term placentas (n = 3 each) of healthy pregnancies as previously described by Lang et al. (2008) and kindly provided by the Department of Obstetrics and Gynecology at the University Hospital Graz. In brief, arterial and venous chorionic blood vessels were resected and separately perfused. The obtained arterial endothelial cells (ECA) and venous endothelial cells (ECV) were centrifuged and the cell pellet was resuspended in EGM-MV medium (Lonza, Verviers, Belgium). Cells were plated on culture plates in a humidified atmosphere at 37°C and 5% CO_2_. Human Hofbauer cells (HBC) were isolated from healthy term placentas (n = 3) as previously described by Schliefsteiner et al. (2020) and kindly provided by the Department of Obstetrics and Gynecology at the University Hospital Graz. In brief, placental villi were washed in buffered saline before digesting the tissue with trypsin, collagenase A and DNase I. Cells were afterwards centrifuged on a Percoll gradient at 1,000 g for 30 min. Hofbauer cells-enriched layers were further purified with negative immune selection, using magnetic beads against CD10 and EGFR. HBCs were then plated in macrophage medium (MaM) supplemented with 5% FCS, macrophage growth supplements (ScienCell, Carlsbad, CA United State) and antibiotics (Pen/Strep, Gibco, Carlsbad, CA, United State) at 37°C.

### Measurement of extracellular ATP

For measurements of extracellular ATP, BeWo cells were cultured in 96-well dishes (Nunc) with a density of 1 × 10^5^ cells/well overnight and afterwards transfected with CD39- and control plasmid, as described above. After 24 h of transfection, the control as well as the CD39-overexpressing cells were treated with pooled platelet releasate (as described above). RealTime-Glo™ Extracellular ATP Assay Reagent was added to the treatment in a final volume of 25% (v/v) to each well and luminescence was measured *via* CLARIOstar^Plus^ (BMG Labtech) every 5 min for 5 h. CD39 overexpression was confirmed by gene expression analysis of *ENTPD1* in each experiment.

### qPCR analysis

Tissue samples and cells were lysed in RNA Lysis Buffer (ExtractMe Total RNA Kit, Blirt, Gdansk, Poland) supplemented with dithiothreitol (DTT, 20 µM). Tissue samples were homogenized with the TissueLyser LT (Qiagen) and Stainless Steel Beads (5 mm, Qiagen). After tissue lysis samples were sonified with a Bioruptor^®^ Pico sonication device (diagenode) for 10 cycles lasting 10 s each at 4°C. Total RNA from lysed tissues and cells was afterwards isolated with the ExtractMe Total RNA Kit (Blirt, Gdansk, Poland) according to the manufacturer´s protocol. Amount calculation and quality check was performed by Nanodrop (ND-1000, Peqlab Biotechnology GmbH; Erlangen, Germany) followed by reverse transcription of 1–2 µg total RNA per reaction using High-Capacity cDNA Reverse Transcription Kit (Applied Biosystems, Foster City, CA, United State). qPCR was performed with SYBR Green qPCR Kit (Biozym, Vienna, Austria) using a Bio-Rad CFX384 Touch Real-Time PCR Detection System (Bio-Rad; Hercules, CA, United State). Used primers are shown in Supplementary Table S1. Fetal sex of first trimester placenta samples was determined with specific primers for DDX3Y and for XIST (Nonn et al., 2021; Nonn et al., 2019). Ct values and relative quantification of gene expression were automatically generated by the CFX Manager 3.1 Software (Bio-Rad Laboratories; Hercules, CA, United State) using the expression of three reference genes YWHAZ, TBP or GAPDH, as recently recommended for gene expression analysis in human placental tissue (Drewlo et al., 2012).

### Immunoblot

Tissue or cells were washed with PBS and lyzed in RIPA buffer (Sigma-Aldrich) including protease inhibitor cocktail (Roche Diagnostics; Mannheim, Germany) and phosSTOP (Roche Diagnostics). Tissue samples were further homogenized with the TissueLyser LT (Qiagen) and Stainless Steel Beads (5 mm, Qiagen). After tissue lysis samples were sonified with a Bioruptor^®^ Pico sonication device (diagenode) for 10 cycles lasting 10 s each at 4°C. Tissue and cell lysates were centrifuged at 8,000 rpm and 4°C for 10 min. Total protein concentration was determined in clear supernatants using the Lowry method. 20 μg total protein per sample were loaded to 10% Bis-Tris gels (NuPAGE, Novex; lifetechnologies) and after gel electrophoresis, proteins were blotted on a 0.45 µm nitrocellulose membrane (Hybond, Amersham Biosciences, GE Healthcare Life Sciences, Little Chalfont, United Kingdom). Ponceau staining (Ponceau S solution, Sigma Aldrich) of the membranes determined blotting efficiency. Primary antibodies, summarized in Supplementary Table S2, were incubated on membranes overnight at 4°C, before HRP conjugated goat anti-rabbit IgG (Bio-Rad, 1:5,000) or HRP conjugated goat anti-mouse IgG (Bio-Rad; 1:5,000) were used as a secondary antibody. After an incubation on membranes for 2 h at RT, immunodetection was performed with a chemiluminescent immunodetection kit (WesternBright Chemilumineszenz Substrat für Filn, Biozym) according to the manufacturer´s instructions. Images were acquired with iBright CL 1000 Imaging System (Thermo Fischer Scientific) and band densities were analyzed with Image Studio Lite 5.2 (Guettler et al., 2021). Results are presented as a ratio of target protein and β-actin or GAPDH band densities.

### Immunohistochemistry

Human formalin-fixed paraffin-embedded (FFPE) placenta tissue from first and third trimester was cut (5 μm) and mounted on Superfrost Plus slides (Menzel-Gläser, Thermo Scientific). Deparaffinization in HistoLab-Clear and a descending alcohol row was followed by antigen retrieval in the multifunctional microwave tissue processor KOS in Epitope Retrieval Solution pH 9.0 (Novocostra, Leica) for 15 min at 93°C. Thereafter, immunohistochemistry was performed using the UltraVision Large Volume Detection System HRP Polymer Kit (Thermo Fisher Scientific) as previously described by Guettler et al. (2021). Anti-CD39 recombinant rabbit monoclonal antibody (JA90-36, Thermo Scientific; 1:1,000) and anti-CD73 rabbit monoclonal antibody (D7F9A, Cell Signaling; 1:200) were used as primary antibodies. Nuclei were stained with Mayer’s hemalaun (Thermo Scientific) and afterwards slides were mounted with Kaiser’s glycerol gelatine (Merck). For negative controls, adjacent slides from serial sections were incubated with Negative Control for Rabbit Immunoglobulin Fraction (Dako, Agilent; 1:7,500). The slides were analyzed with an Olympus microscope (BX3-CBH).

### Statistics

Data were analyzed using GraphPad Prism Version 9.2.0 and are presented as mean values with standard error of mean (SEM). Data were tested for normal distribution using the Shapiro-Wilk test and in case of non-normality, significance between two groups was tested with the two-tailed Mann Whitney U test. Outliers were identified using Grubbs test (α = 0.05). Linear regressions were calculated in R (v4.2.2, 64-bit) using the main package tidyverse v2.0.0. A *p*-value of less than 0.05 was considered statistically significant.

## Results

### Developmental changes in placental tissue expression of CD39 and CD73

**FIGURE 1:**
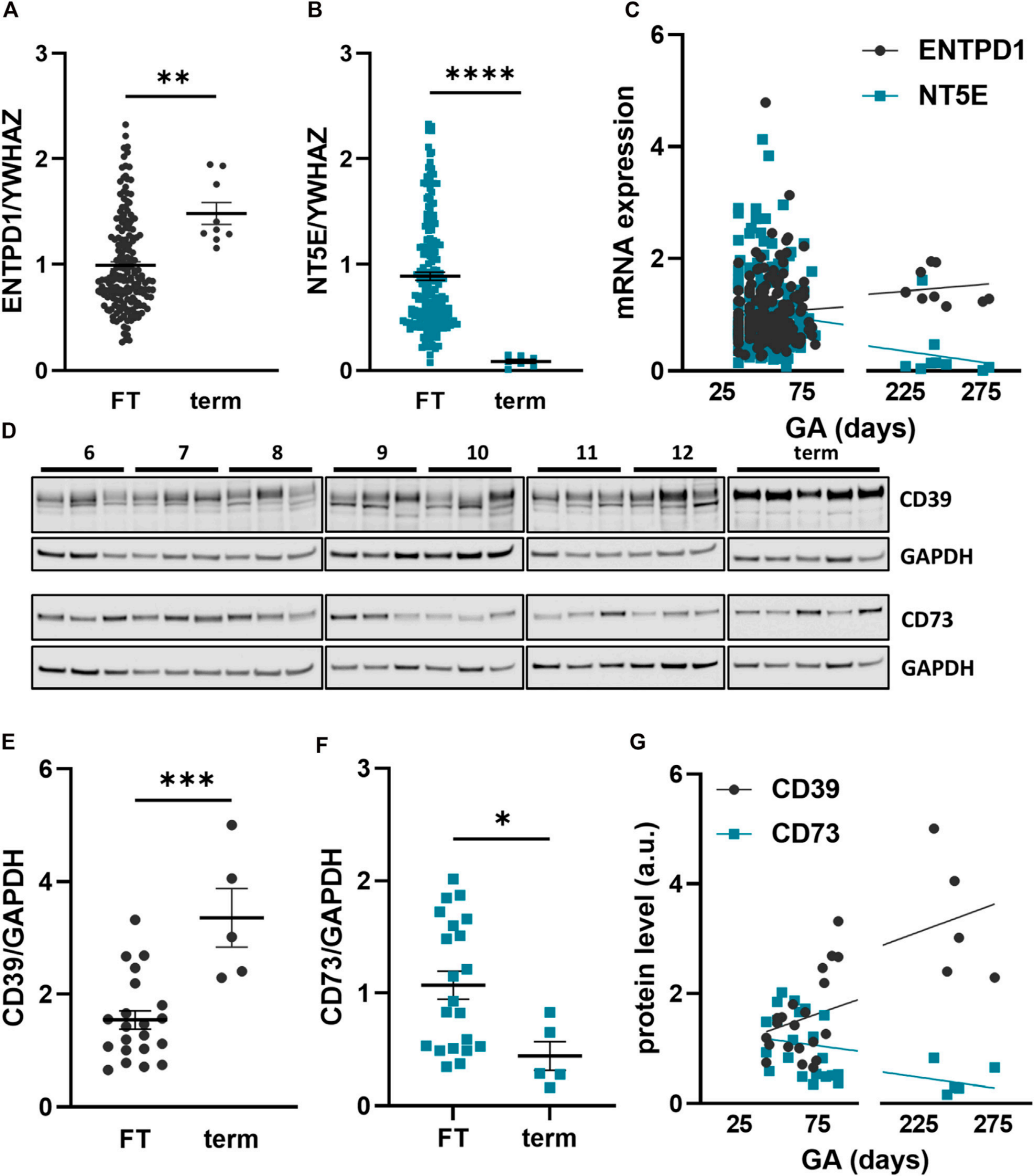
Placental CD39 and CD73 expression over human gestation. Expression of CD39, encoded by *ENTPD1*, and CD73, encoded by *NT5E*, was analyzed in first trimester (FT) as well as third trimester (term) placental tissue on mRNA **(A–C)** and protein level **(D–G)**. CD39 is increasing towards term on gene expression (FT n = 186, mean gestational week 7.72 ± 1.7 vs. term n = 9, mean gestational week 35.48 ± 2.64) **(A, C)** and on protein level (FT n = 21, mean gestational week 9.27 ± 2.15 vs. term n = 5, mean gestational week 35.89 ± 2.18) **(D, E, G)**. Gene expression **(B, C)** and protein levels **(D, F, G)** of CD73 are decreasing at term of pregnancy. Immunoblotting for placental CD39 and CD73 (**D**). Band density of CD39 and CD73 was normalized to GAPDH. *YWHAZ* was used as reference gene. Data are presented as mean ± SEM. *p ≤ 0.05, **p ≤ 0.01, ***p ≤ 0.0002, ****p ≤ 0.0001.

**FIGURE 2:**
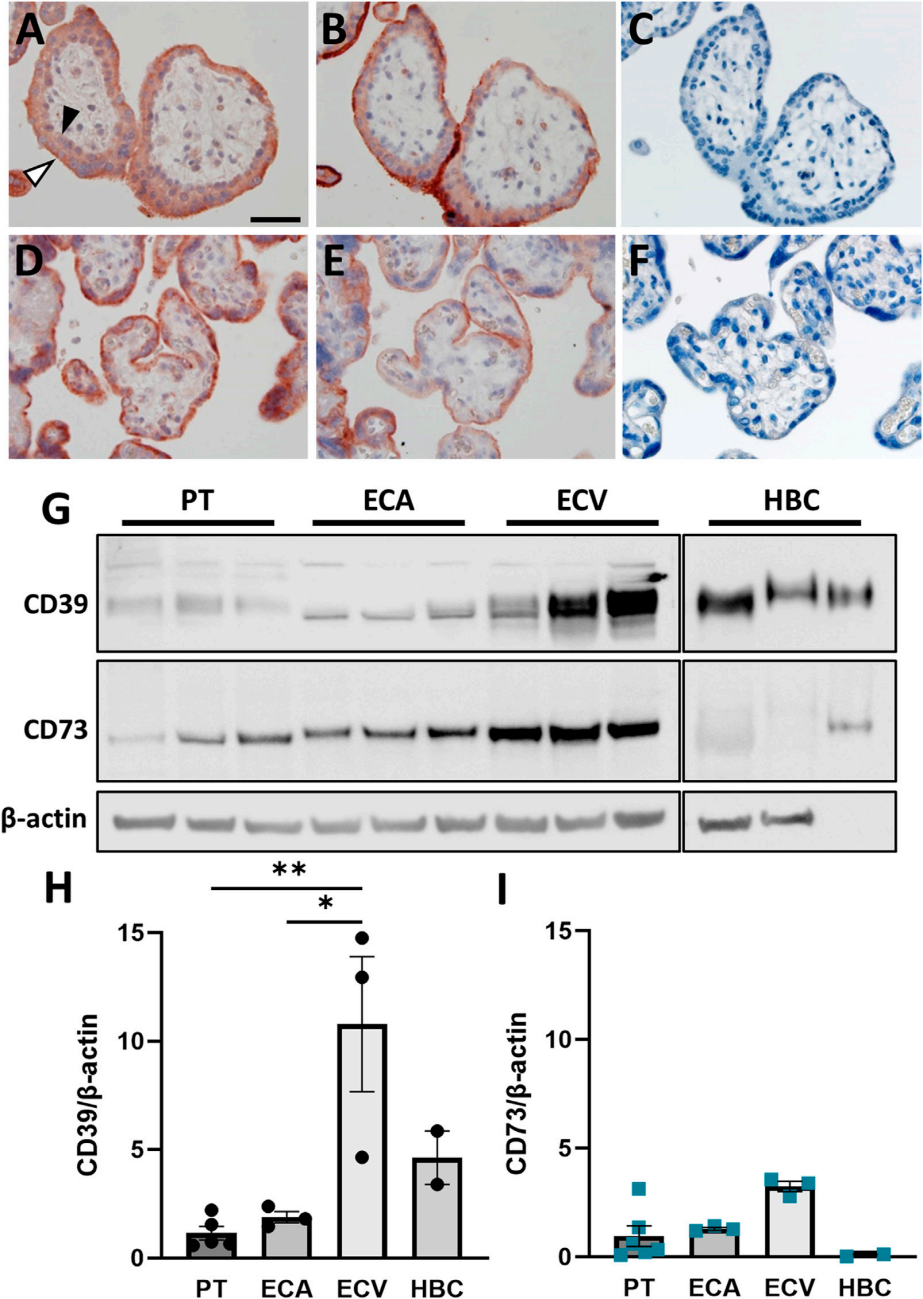
CD39 and CD73 are expressed in key placental cell types. Serial sections of human first trimester (**A, B,** GA 7 + 5) and term (**D, E,** GA 39 + 0) placental villous tissue were subjected to immunochistochemistry. CD39 staining **(A, D)** revealed positive signals on cytotrophoblasts (black arrowhead) as well as on the syncytiotrophoblast, with higher intensity towards the apical side (white arrowhead). Positive staining for CD73 **(B, E)** was only found in the syncytiotrophoblast. Negative control for rabbit immunoglobulin fraction gave no staining on first trimester **(C)** and term placental tissue **(F)**. Scale bar represents 50 μm. CD39 and CD73 was investigated in primary term trophoblast (PT, n = 6), arterial endothelial cells (ECA, n = 3), venous endothelial cells (ECV, n = 3) and Hofbauer cells (HBC, n = 3) from healthy term pregnancies **(G)**. CD39 and CD73 were detected in all cell types, but predominantly in ECVs. Band densities of CD39 **(H)** and CD73 **(I)** were normalized to β-actin. Data are presented as mean ± SEM. *p ≤ 0.05, **p ≤ 0.01.

In order to elucidate developmental changes of placental CD39 and CD73 over gestation, expression was analyzed in placental tissue at different stages of pregnancy. Within the first trimester, placental CD39 expression encoded by *ENTPD1* was unaltered (*R* ^2^ = 0.003, *p* = 0.44; linear regression of samples ≤84 days gestation), while it significantly increased 1.5-fold in term placenta compared to the first trimester tissue (Figure 1A). Like CD39, placental CD73 (encoded by *NT5E*) expression showed no changes throughout the first trimester (*R* ^2^ = 0.01, *p* = 0.18), but significantly declined 10.2-fold at term (Figure 1B). Linear regression analysis of all samples (gestational days 35–280) confirmed a significant increase in placental *ENTPD1* expression (*R* ^2^ = 0.03, *p* = 0.02), while at the same time *NT5E* expression levels significantly declined with gestational age (*R* ^2^ = 0.05, *p* = 0.006; Figure 1C). Furthermore, analyses of maternal smoking during first trimester, fetal sex, maternal age, and body mass index (BMI) did not reveal any effects on placental *ENTPD1*. None of the above parameters, except maternal BMI (*R* ^2^ = 0.02, *p* = 0.03), correlated with NT5E expression. However, correlation of placental NT5E expression and gestational age remained significant even after correction for maternal BMI. On protein level, placental CD39 increased 2.3-fold at term compared to first trimester (Figures 1D,E), while CD73, in contrast, decreased 2.43-fold (Figures 1D,F). In line with mRNA expression data, linear regression analysis of protein levels showed a significant increase of placental CD39, together with a decrease in placental CD73 over gestation (Figure 1G). Immunohistochemistry of human placenta tissue sections detected both CD39 and CD73 predominantly in the syncytiotrophoblast layer. In first trimester, staining of both ectonucleotidases seemed most intense at the microvillous plasma membrane of the syncytiotrophoblast (Figures 2A,B), whereas the villous cytotrophoblast population was only positive for CD39. At term, both ectonucleotidases were located at the syncytiotrophoblast, with CD39 (Figure 2D) appearing slightly stronger and CD73 (Figure 2E) much weaker than in the first trimester. In order to reveal the distribution of both ectonucleotidases in primary placental key cell types, including term trophoblasts, arterial endothelial cells, venous endothelial cells and Hofbauer cells were analyzed on protein level *via* immunoblot for CD39 and CD73 (Figures 2G–I). Both ectonucleotidases were found in term PTs, ECAs, ECVs and HBCs with highest abundance in venous endothelial cells. Overall protein levels of CD73 were generally lower than CD39 in all analyzed cell types (Figures 2G–I). In summary, these data suggest that CD39 and CD73 are differentially expressed in human placenta over gestation.

### Placental CD39 and CD73 are upregulated in PE cases

**FIGURE 3:**
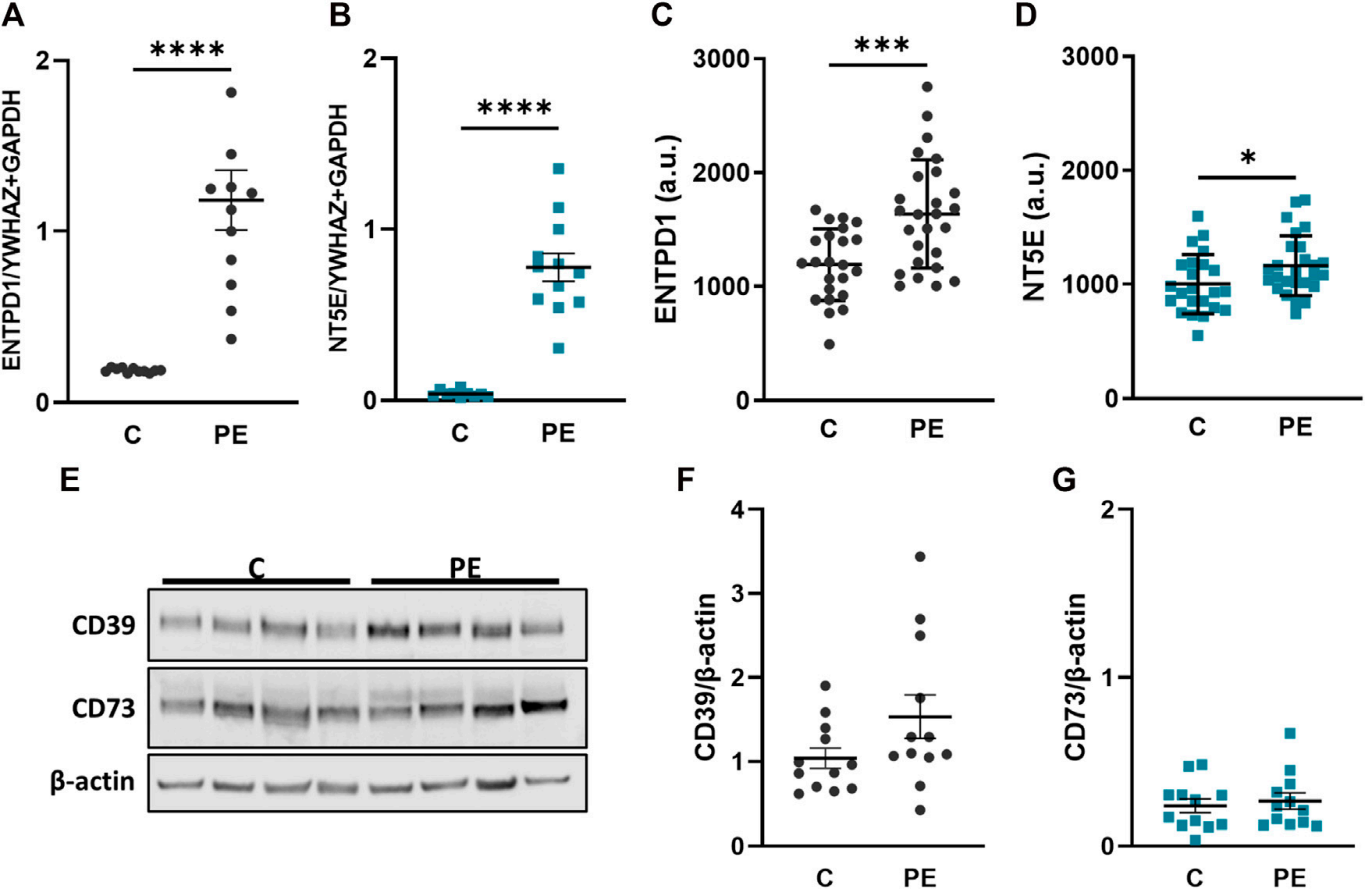
CD39 is upregulated in preeclampsia. Placental villi from healthy (C, n = 12, mean gestational week 38.64 ± 0.56) and preeclamptic (PE, n = 12, mean gestational week 35.34 ± 3.70) patients were subjected either to gene expression **(A, B)** or immunoblot analyses **(E–G)**. *ENTPD1* **(A)** and *NT5E* **(B)** were significantly upregulated in preeclamptic cases compared to controls. *ENTPD1 and NT5E* were also analyzed from a previously published microarray dataset (Herse et al., 2012) in controls (n = 23) and PE (n = 26) cases **(C, D)**. Immunoblot analysis of CD39 **(E, F)** showed a slight increase in preeclamptic cases, whereas protein analysis of CD73 **(E, G)** revealed no difference between C and PE. Protein levels were normalized to β-actin. *YWHAZ* and *GAPDH* were used as reference genes for gene expression analysis. Data are presented as mean ± SEM. ****p ≤ 0.0001.

Pregnancy complications, such as preeclampsia, are often accompanied with changes in the coagulation system and therefore we aimed to elucidate whether the ectonucleotidases are dysregulated in placental tissue from preeclampsia cases. CD39 (*ENTPD1*) was significantly upregulated in placental tissue from patients suffering from PE compared to the controls (Figure 3A). The expression of CD73 (*NT5E*) was in general lower than CD39 (*ENTPD1*) in both controls and PE, (Figures 3A,B), but CD73 (*NT5E*) was also significantly increased in PE compared to control cases (Figure 3B). Furthermore, microarray analysis of another, larger placental tissue cohort that included healthy and preeclamptic cases (Herse et al., 2012), confirmed significant upregulation of both ectonucleotidases in preeclamptic tissue compared with controls (Figures 3C,D). At this point it should be noted that the PE groups in both cohorts showed a significantly lower gestational age, compared to controls, which however, may not be the cause of the increased *ENTPD1* (CD39) expression in the PE cases, since its expression is increasing towards term in healthy controls. However, in order to exclude the bias of gestational age, we examined placental *ENTPD1* and *NT5E* expression in a publicly available microarray dataset (Leavey et al., 2016) of early-onset PE cases and age-matched preterm controls, showing results that were in good agreement with our results (Supplementary Figure S2). Data from immunoblot analysis for CD39 confirmed this data, and showed a trend towards upregulation in placenta samples from PE patients compared to controls (Figures 3E,F). Although not directly comparable due to different antibody specificities, CD73 protein levels appeared also lower compared to those of CD39 (Figures 3E–G), but no notable difference between control and PE cases was detected (Figure 3G).

### CD39 in response to platelet-derived factors and different oxygen levels

**FIGURE 4:**
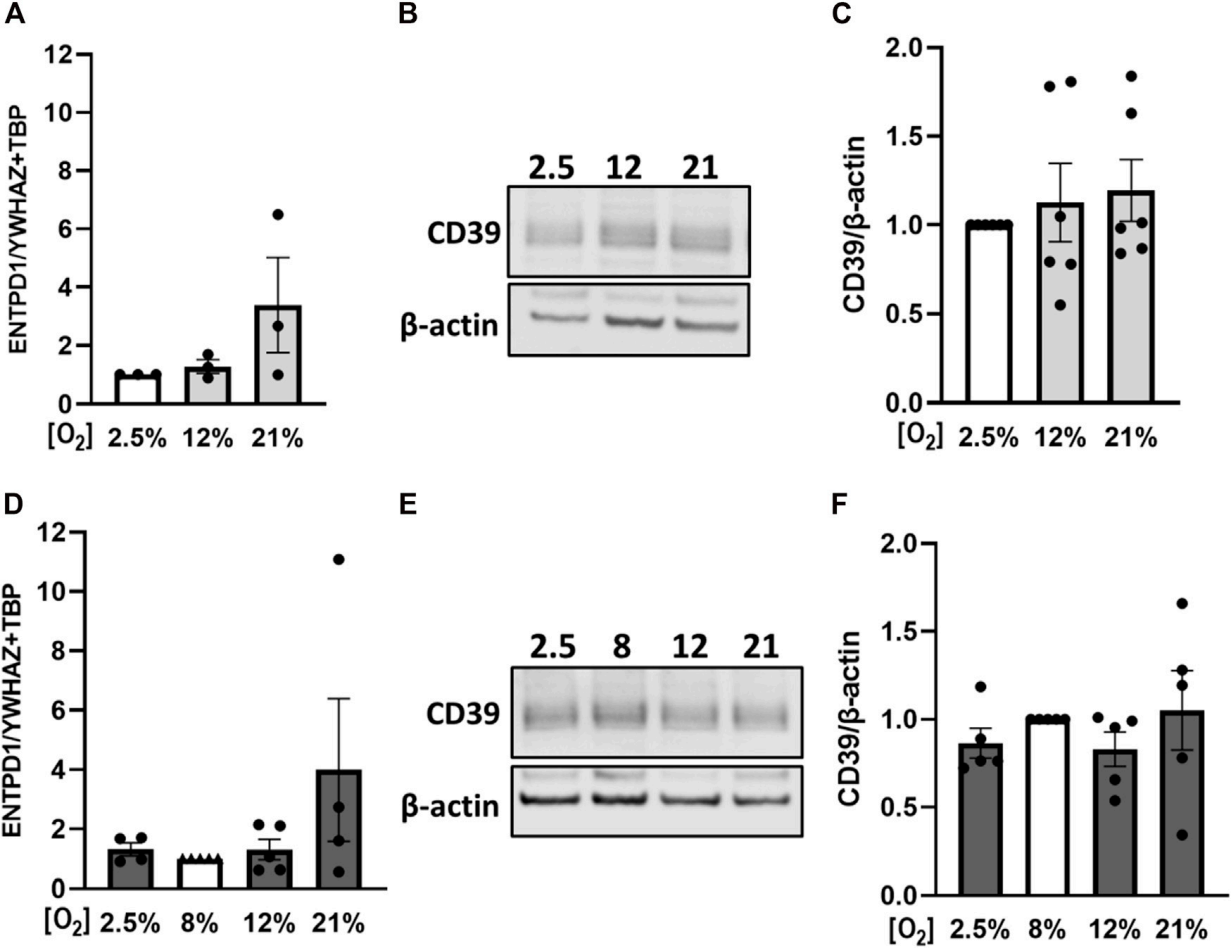
Effect of oxygen on placental CD39 expression. Human first trimester **(A–C)** and term **(D–F)** placental explants were incubated for 24 h under indicated oxygen concentrations. Gene expression analysis of *ENTPD1* in first trimester tissue (n = 3, mean gestational week 7.48 ± 0.91) revealed a trend towards higher expression upon increasing oxygen concentrations **(A)**. Protein levels of CD39 were analyzed *via* immunoblot in first trimester tissue **(B, C)** (n = 6, mean gestational week 7.90 ± 0.78). Healthy term placental explants were analyzed after incubation on mRNA **(D)** as well as on protein **(E, F)** level (n = 5, mean gestational week 38.49 ± 0.54). Values from control samples - first trimester (incubated at 2.5% O_2_, **A–C**) and term placentas (incubated at 8% O_2_, **D–F**)—were set to 1. Band density was normalized to β-actin **(B, C, E, F)**. *YWHAZ* and *TBP* served as reference genes for gene expression analysis **(A, D)**. Data are presented as mean ± SEM.

Since placental CD39 was dysregulated in PE cases, we aimed to analyze the regulation of placental CD39 in response to different oxygen levels and platelet-derived factors. For this purpose, placental villi from early and late pregnancies were first subjected to different oxygen concentrations. Control samples of first trimester placental tissue were incubated at 2.5% O_2_, which represents normal placental oxygen conditions at that early stage of pregnancy (Miller et al., 2005). Hyperoxic conditions, such as 12%, did not change expression levels of CD39, whereas oxygen levels of 21% lead to a trend towards higher expression levels (Figure 4A). On protein level, CD39 showed the same trend towards higher levels with increasing oxygen concentrations (Figures 4B,C). Placental villi from third trimester in contrast, were exposed to hypoxia as well as to hyperoxia, taking 8% as reference for control samples (Miller et al., 2005). Neither 2.5% nor 12% oxygen had a noteworthy effect on CD39 levels in comparison to controls (Figure 4D). However, in response to 21% oxygen, placental CD39 showed a trend towards higher expression levels compared to explants exposed to 8% O_2_ (Figure 4D). On protein level, neither hypoxia nor hyperoxia led to an alteration of CD39 (Figures 4E, F). Of note, it should be taken into account, that oxygen concentrations of 21% are not considered as physiological oxygen concentrations (Figures 4A,D).

**FIGURE 5:**
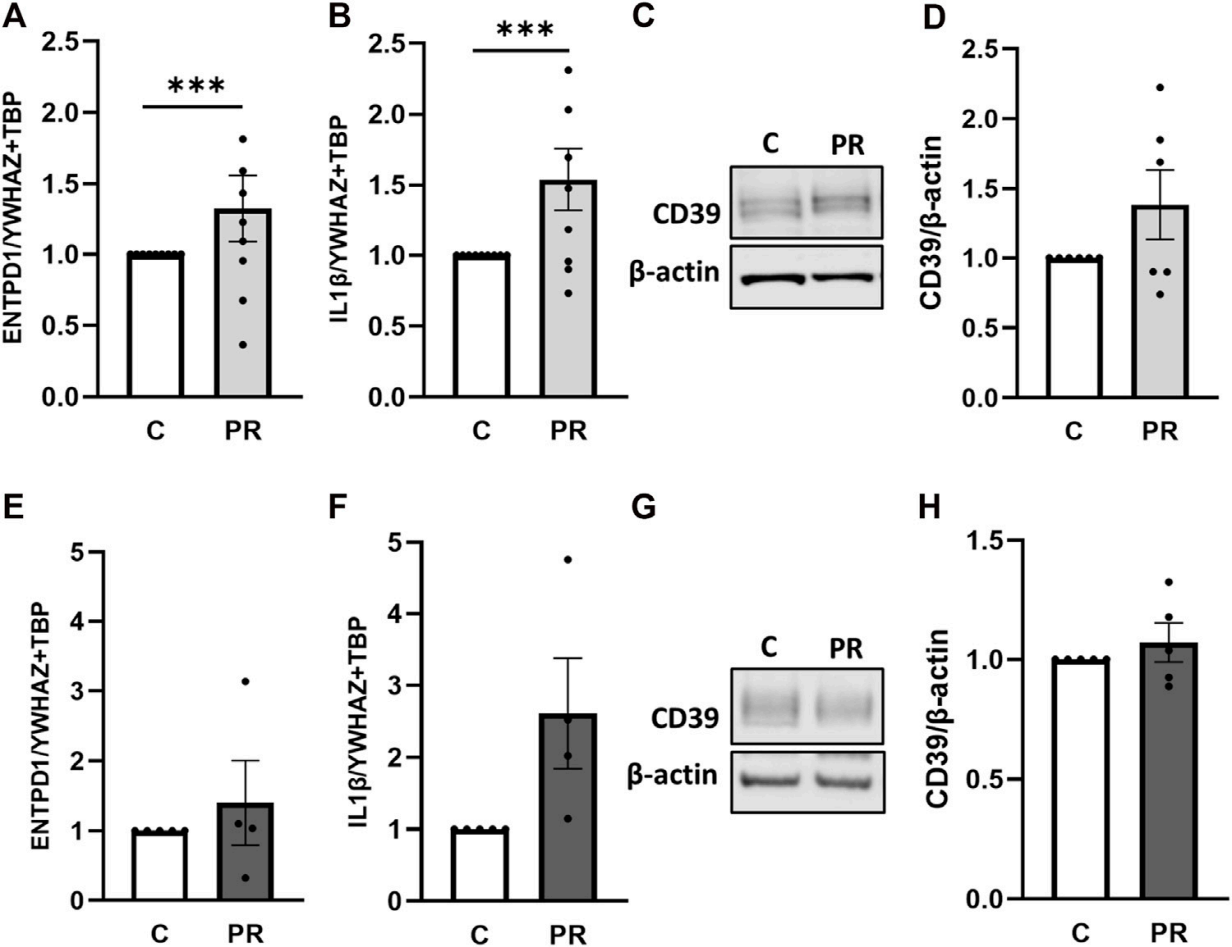
Platelet-derived factors induce placental IL-1β expression. Placental explants from first trimester **(A–D)** and term **(E–H)** were incubated for 24 h in presence or absence of a platelet releasate (PR; pool of 10 healthy pregnant women at term). First trimester explants were incubated at 2.5% O_2_ **(A–D)**, whereas placental villi from third trimester were incubated at 8% O_2_ **(E–H)**. After incubation with platelet releasate, placental villi from first trimester (**A, B**, n = 9; mean gestational week 7.66 ± 0.88) and term (**E, F**, n = 5; mean gestational week 38.49 ± 0.54) were subjected to gene expression analysis for *ENTPD1* and *IL-1β*. Immunoblot analysis revealed a trend towards higher levels of CD39 upon platelet releasate treatment in early (**C, D**; n = 6; mean gestational week 7.90 ± 0.78) and late (**G, H**; n = 5; mean gestational week 38.49 ± 0.54) cases. Band density was normalized to β-actin **(C, D, G, H)**. *YWHAZ* and *TBP* served as reference genes for gene expression analysis **(A, B, E, F)**. Data are presented as mean ± SEM. ***p ≤ 0.0002.

Next, we aimed to analyze the impact of platelet releasates from healthy pregnant women on placental CD39 expression. Accordingly, gene expression analysis of CD39 in first trimester tissue increased 1.33-fold upon treatment with platelet-derived factors (Figure 5A). At the same time, levels of IL-1β, described to be released upon inflammasome activation in trophoblasts by Kohli et al. (2021), increased 1.54-fold in response to platelet-derived factors (Figure 5B). Immunoblot analysis for CD39 also showed a slight increase upon treatment with platelet releasate compared to controls (Figures 5C,D). Gene expression analysis of term placental tissue *ex vivo* cultures revealed a trend towards higher expression levels of CD39 (Figure 5E) in response to platelet-derived factors. Like for first trimester, analysis of IL-1β revealed a 2.6-fold increase under influence of platelet-derived factors in term placental explants (Figure 5F). On protein level, no effect of platelet-derived factors on placental CD39 was detected (Figures 5G,H).

### Trophoblastic CD39 overexpression increases hydrolysis of extracellular ATP and abrogates platelet-derived factors-induced IL-1β expression

**FIGURE 6:**
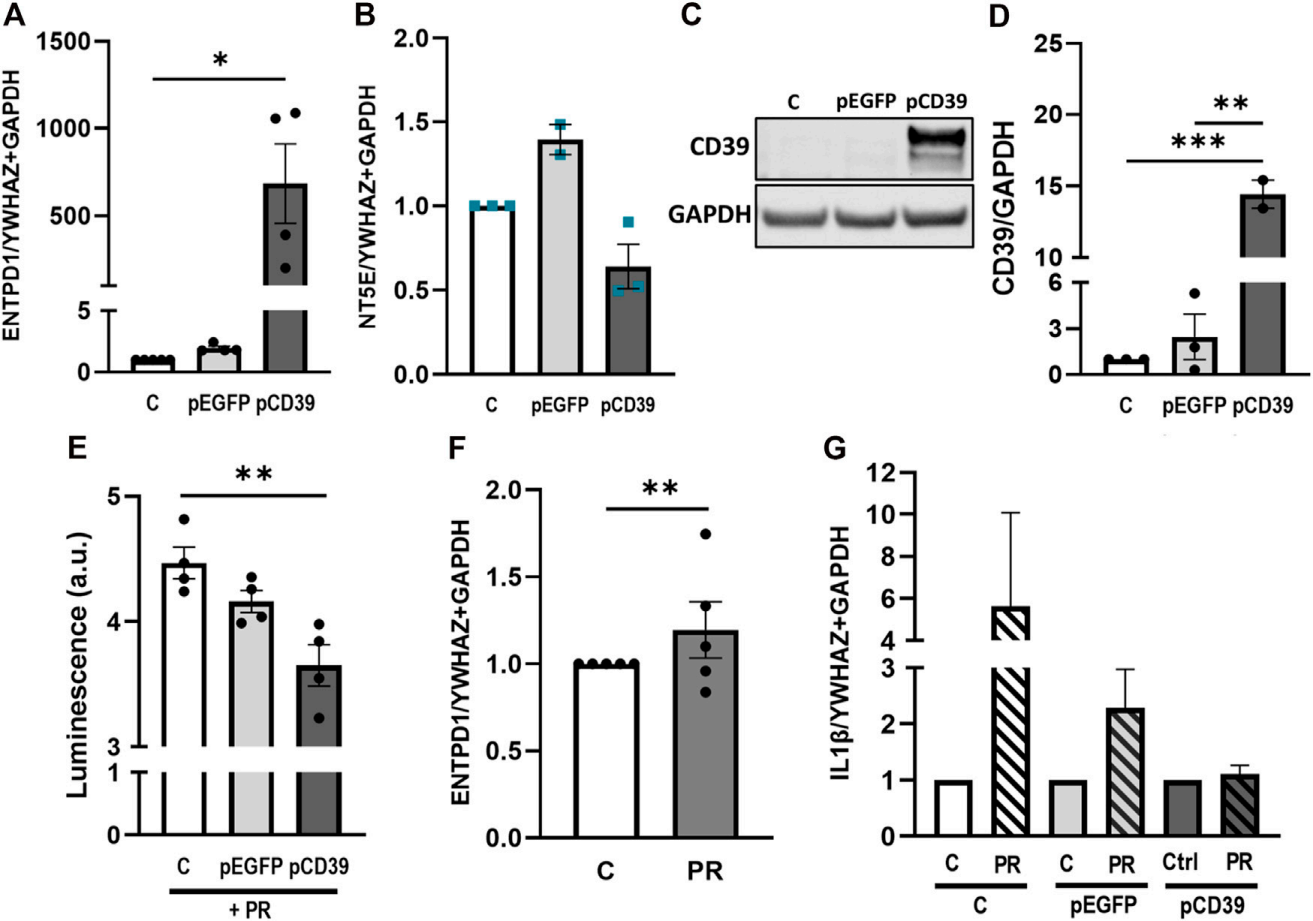
Platelet releasate-induced IL-1β upregulation is abrogated by CD39 overexpression. BeWo cells were transfected with pCD39 or an empty vector for 24 h and were afterwards subjected to qPCR and protein analysis **(A–D)**. Gene expression analysis of *ENTPD1* revealed a strong upregulation in pCD39-transfected samples **(A)**. *NT5E* was not significantly affected by CD39 overexpression **(B)**. Immunoblot analysis confirmed CD39 overexpression in BeWo cells **(C, D)**. Band density was normalized to GAPDH **(C, D)**. Extracellular ATP levels in CD39 overexpressing and control BeWo cell cultures were measured in presence of platelet releasate (PR; pool of 10 healthy pregnant women at term) for 5 h *via* luminescence measurement **(E)**. BeWo cells were incubated with platelet releasate for 24 h and subsequently subjected to qPCR of endogenous *ENTPD1* **(F)**. CD39 was overexpressed in BeWo cells for 24 h and cells afterwards incubated in presence or absence of platelet releasate for another 24 h. Cells were subjected to gene expression analysis for IL-1β **(G)**. For gene expression analysis *YWHAZ* and *GAPDH* were used as reference genes. Data are presented as means ± SEM from three **(B–D)**, four **(E)**, and five **(A, F, G)** independent experiments. *p ≤ 0.05, **p ≤ 0.01, ***p ≤ 0.0002.

In order to elucidate the mechanistic relevance of placental CD39 in pro-inflammatory conditions, we next overexpressed CD39 in BeWo cells and cultured them in presence or absence of platelet releasates. Transfection efficiency was verified on gene expression and protein level (Figures 6A,C,D), showing massive overexpression of CD39 when compared to controls. Of note, endogenous CD73 (*NT5E*) was slightly but not significantly decreased in CD39-overexpressing cells (Figure 6B). Kinetic measurements of extracellular ATP revealed that overexpression of CD39 in trophoblastic cells cultured in presence of platelet-derived factors, led to a significant decrease of extracellular ATP compared to control cells (Figure 6E). Platelet releasates did not influence the endogenous CD39 expression of BeWo cells (Figure 6F). However, platelet releasate-induced upregulation of IL-1β was almost restored to control levels in BeWo cells overexpressing CD39 (Figure 6G).

## Discussion

This study demonstrates the expression dynamics of the placental ectonucleotidases CD73 and CD39 over a broad time range of human pregnancy. While CD73 decreases in the placenta as pregnancy progresses, CD39, in contrast, steadily increases, indicating an increasing demand for extracellular ATP clearance at the utero-placental interface during the late phase of pregnancy. Moreover, our data from three different placental tissue cohorts show that placental CD39 is increased in preeclampsia, a pregnancy disease associated with 2.5-fold enhanced plasma ATP levels in affected women (Bakker et al., 2007). Elevated plasma ATP is thought to induce a pro-inflammatory response that not only leads to glomerular albuminuria, as previously shown by ATP infusion into pregnant rats (Faas et al., 2010; Spaans et al., 2014), but may also trigger purinergic receptor-mediated NLRP3 inflammasome activation in the placental trophoblast (Kohli et al., 2016; Kohli and Isermann, 2017). We observed an increase of IL-1β when trophoblasts were exposed to platelet releasates, suggesting placental inflammasome activation as a consequence of platelet activation. Importantly, induction of IL-1β could be abrogated in trophoblasts by CD39 overexpression, suggesting CD39 as an important defense mechanism against placental thrombo-inflammation.

In general, CD39 is expressed on platelets, leukocytes, and the vascular endothelium, representing the dominant ectonucleotidase in the vasculature, where it plays major roles in controlling the immune response, vascular inflammation and thrombosis (Morello et al., 2021). In human pregnancy, around transition to the second trimester, the placenta is fully perfused with maternal blood. This utero-placental blood passage is enabled by connecting maternal blood vessels to the so-called intervillous space of the placenta, which is entirely lined by the syncytiotrophoblast. Hence, CD39 on the surface of placental villi [accounting for approximately 10 m^2^ at term (Biswas et al., 2008)] may be considered to fulfill similar functions as described for the vascular endothelium. As the rate-limiting enzyme in ectonucleotidase-mediated ATP to adenosine conversion, CD39 is the molecular break in regulating extracellular ATP, which functions as a danger signal (DAMP), triggering activation of P2 receptors and downstream pro-inflammatory responses (Kanthi et al., 2014; Zhao et al., 2017; Willcox et al., 2022). The CD39/CD73 hydrolytic activity on the purinergic cascade is an important immunoregulatory effector, by the breakdown of ATP into the anti-inflammatory molecules ADP and AMP and finally into adenosine (da Silva et al., 2022). Increased levels of adenosine in an acute response manner are intended to be beneficial because of the activation of anti-inflammatory and tissue-protective pathways (Zhou et al., 2009), but when it comes to chronically elevated adenosine levels in persistent inflammatory conditions, it contributes to pro-inflammatory pathways (Zhou et al., 2009). Increased adenosine signaling in the placenta is suggested to be linked to the pathogenesis of preeclampsia (Iriyama et al., 2015). It has been shown that circulating adenosine concentrations, as a result of increased ATP release, are elevated in women suffering from preeclampsia (von Versen-Höynck et al., 2009) and that in preeclampsia, maternal adenosine concentration correlates with the severity of the syndrome (Yoneyama et al., 2002b).

Our findings of elevated placental CD73 in preeclampsia patients are in line with a study from Iriyama et al., who also showed significantly increased mRNA levels of CD73 in preeclamptic placenta tissue. They also showed an increase of CD39 mRNA levels in preeclampsia patients, which was however not significant. Furthermore, they found increased CD73 protein levels and increased enzymatic activity of CD73 in preeclampsia patients and concluded that elevated CD73 levels are responsible for increased placental adenosine (Iriyama et al., 2015). They furthermore showed in an experimental preeclampsia mouse model, which was established by the transfer of preeclampsia patient-derived IgG, that placental CD73 activity and adenosine levels were significantly increased in preeclampsia-IgG-injected mice (Iriyama et al., 2015). It should be noted at this point that NTPDases are described to undergo several post-translational modifications affecting both tertiary and quaternary structure, which may affect their enzymatic function (Atkinson et al., 2006). Hence, expression levels may not necessarily correlate with NTPDase expression levels.

Analyses of key placental cell types, isolated from term placenta, suggests expression of both ectonucleotidases in villous trophoblasts and fetal-placental endothelial cells, with venous endothelial cells showing higher expression levels than those of the arterial vasculature. This is not surprising, since placental arterial- and venous endothelial cells show considerable differences in their phenotype and genotype (Lang et al., 2008; Lassance et al., 2012). With exception of the lung, our finding of differential ectonucleotidase expression in the arterial- and venous endothelium may not be directly translated to other human organs. In the human placenta, arterial endothelial cells are exposed to less oxygen tension than venous endothelial cells, as deoxygenated blood is pumped from the fetal heart through two umbilical arteries *via* villous arteries to terminal villi. Here, the blood is oxygenated at the so-called vasculosyncytial membranes and afterwards is transported through villous veins and one umbilical vein back to the fetus (Nodwell et al., 2005). However, there are reports of differential endothelial CD39 expression that rather exclude oxygen as a major regulator. A recent study showed a significant enrichment of *ENTPD1* mRNA in human saphenous vein endothelial cells, when compared with human aortic- and coronary arterial endothelial cells (Maier-Begandt et al., 2021). Since saphenous veins, unlike fetal placental veins, transport oxygen-poor blood, regulatory mechanisms other than oxygen must be involved in controlling endothelial CD39 expression. Regardless of its regulation, it is tempting to speculate on an increased need for CD39-mediated protection against thromboembolic vascular injury in the venous- than the arterial compartment of the placenta. Indeed, mural and occlusive thrombi can often be detected in the superficial placental vessels and their branches, and are more commonly found in the veins (Baergen, 2005).

Our results from oxygen experiments exclude a major regulatory role of oxygen on placental CD39. This is in contrast with previous studies demonstrating that mRNA- and protein levels, as well as the enzymatic function of CD39 are increased during ischemia, inflammation, or hypoxia (Köhler et al., 2007; Ruan et al., 2022). For example, studies in a model of murine myocardial ischemia/reperfusion show a time-dependent induction of cardiac CD39 mRNA under the control of SP1 (Eltzschig et al., 2009). This discrepancy may be explained by a different tissue context or —in case of *in vitro* studies—by the use of different cell types, such as hepatocytes, and a rather harsh hypoxic setting of 0.1% O_2_ (Li et al., 2017). Of note, cytokine-induced killer cells, a heterogeneous T cell population obtained by *in vitro* differentiation of peripheral blood mononuclear cells, as well as lung adenocarcinoma cell lines show no significant differences in CD39 expression regardless of being cultured either under 21% or 1% O_2_ (Horenstein et al., 2018; Giatromanolaki et al., 2020). However, the physiological relevance of data obtained from our “hyperoxia” results should be interpreted with caution. As mentioned, intrauterine oxygen concentration are relatively low depending on the gestational age, but are expected at 2.5% O_2_ for the first trimester and 8% O_2_ for the third trimester (Miller et al., 2005). Oxygen levels of up to 21%, as shown in our results, are therefore considerably higher than *in vivo* concentrations.

We found no effect of maternal smoking, fetal sex, maternal age, and BMI on placental CD39 expression. The fact that direct- and second-hand cigarette smoke exposure leads to significant deregulation of CD39 in the lung tissue of mice and rats (Kratzer et al., 2012; Lazar et al., 2016), suggests that combustion products of tobacco directly affect CD39 in respiratory epithelial cells, whereas compounds crossing the air-blood barrier of the lung do not affect placental CD39. Data on sex-dependent differences in CD39 expression is very limited, but suggest that antenatal dexamethasone treatment induces sex-specific upregulation of CD39 in rat brains more prominently in males (Manojlovic-Stojanoski et al., 2022).

In conclusion, our study suggests dynamical changes of CD39 and CD73 over the course of gestation. While CD39 is constantly increasing towards term, CD73 decreases until the end of pregnancy. Upregulation of placental CD39 in preeclampsia, suggests an increasing demand for extracellular ATP hydrolysis at the utero-placental interface. Hence, increased placental CD39 may represent a defense mechanism to prevent a trophoblastic inflammasome activation and the subsequent release of the pro-inflammatory cytokine IL-1β.

## Data Availability

The original contributions presented in the study are included in the article/Supplementary Material, further inquiries can be directed to the corresponding author.
