# Supplementary figures and images for: CD39 abrogates platelet-derived factors induced IL-1β expression in the human placenta
Source: Front Cell Dev Biol. 2023 May 30;11:1183793. doi: 10.3389/fcell.2023.1183793 (PMC10264854; doi:10.3389/fcell.2023.1183793)

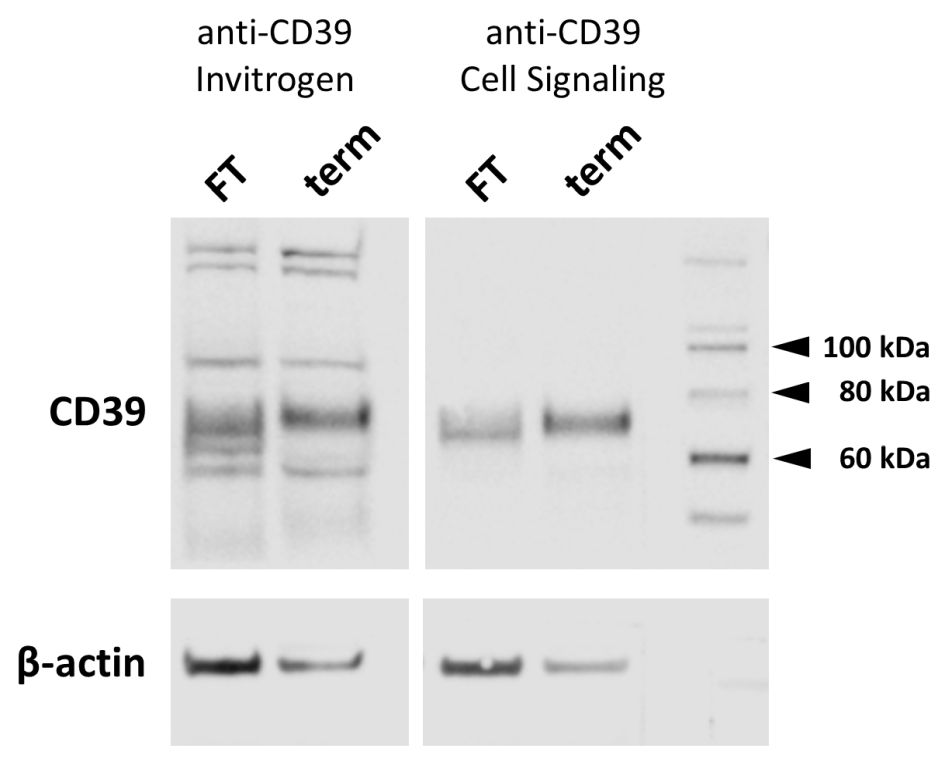

Supplement: Supplementary file 1 [file Image3.TIF]

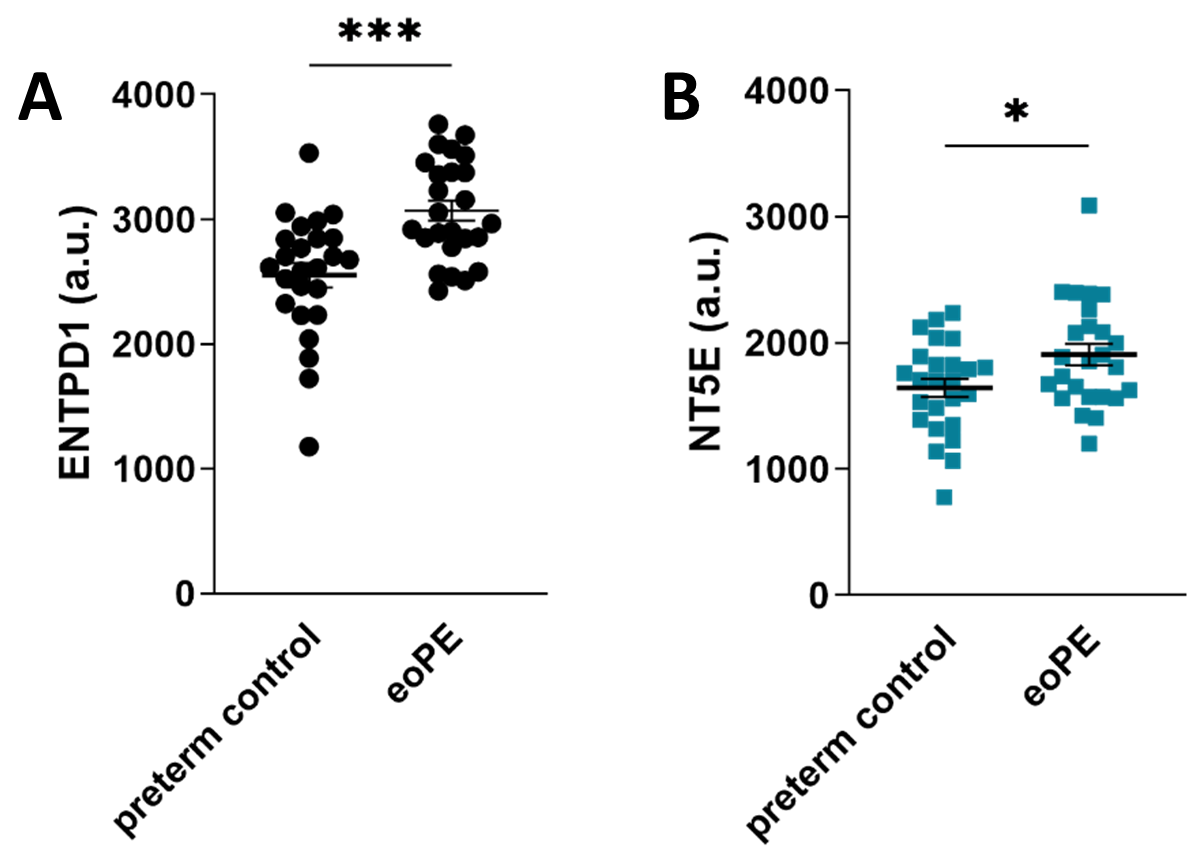

Supplement: Supplementary file 2 [file Image2.TIF]

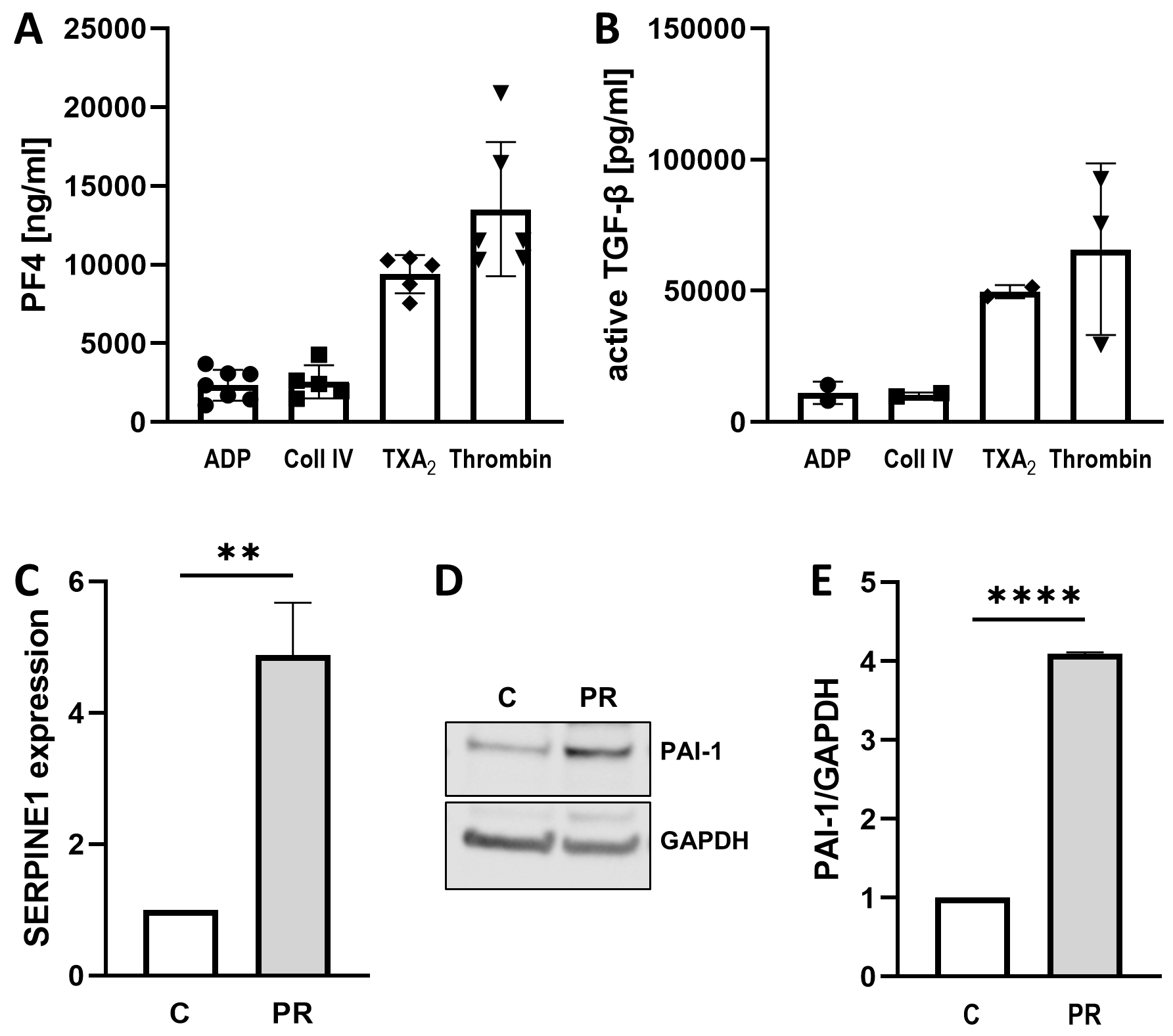

Supplement: Supplementary file 3 [file Image1.TIF]
