# Supplementary material for: CD39 abrogates platelet-derived factors induced IL-1β expression in the human placenta
Source: Front Cell Dev Biol. 2023 May 30;11:1183793. doi: 10.3389/fcell.2023.1183793 (PMC10264854; doi:10.3389/fcell.2023.1183793)
Supplement: Supplementary file 4 [file DataSheet1.pdf]

Supplemental material to:

CD39 abrogates platelet-derived factors induced IL-1β expression in the human placenta

Désirée Forstner[1], Jacqueline Guettler[1], Beatrice A. Brugger[1], Freya Lyssy[1], Lena Neuper[1], Christine Daxboeck[1], Gerhard Cvirn[2], Julia Fuchs[3], Kristin Kräcker[4, 7], Alina Frolova[4, 5, 8], Daniela S. Valdes[4, 5], Christina Stern[6], Birgit Hirschmugl[6], Herbert Fluhr[6], Christian Wadsack[6], Berthold Huppertz[1], Olivia Nonn[1, 4], Florian Herse[4, 5], Martin Gauster[1]

[1]Division of Cell Biology, Histology and Embryology; Gottfried Schatz Research Center, Medical University of Graz; Austria
[2]Division of Medicinal Chemistry, Otto Loewi Research Center, Medical University of Graz, Austria
[3]Division of Medical Physics and Biophysics, Gottfried Schatz Research Center, Medical University of Graz, Austria
[4]Experimental and Clinical Research Center, a cooperation between the Max-Delbrück-Center for Molecular Medicine in the Helmholtz Association and the Charité - Universitätsmedizin Berlin, Berlin, Germany
[5]Max-Delbrück-Center for Molecular Medicine in the Helmholtz Association (MDC), Berlin, Germany
[6]Department of Obstetrics and Gynecology, Medical University of Graz; Austria
[7]Charité – Universitätsmedizin Berlin, corporate member of Freie Universität Berlin and Humboldt-Universität zu Berlin, Berlin, Germany
[8]Institute of Molecular Biology and Genetic of NASU, Kyiv, Ukraine

*Correspondence:
Martin Gauster, https://orcid.org/0000-0003-0386-6857
Division of Cell Biology, Histology and Embryology,
Gottfried Schatz Research Center,
Medical University of Graz,
Neue Stiftingtalstraße 6, Graz 8010, Austria
Tel: +43 316 385 71896, Fax: +43 316 385 79612
e-mail: martin.gauster@medunigraz.at

Supplemental figure legends

Supplemental Figure 1

Efficiency of thrombin-induced platelet activation and confirmation of platelet releasate activity

Platelets from healthy donors were isolated and activated with either ADP (10 µM), Collagen Type IV (20 µg/ml), Thromboxane A2 (2 µM) or Thrombin (1U/ml). PF4 (**A**) and TGF-β (**B**) were measured in platelet releasate by ELISA. Activity of platelet releasate (PR) was tested in BeWo cells by its ability to induce TGF-β downstream target plasminogen activator inhibitor (PAI-1, encoded by SERPINE1). BeWo were seeded overnight and afterwards incubated in presence or absence of PR, which was obtained by thrombin-activation of isolated platelets from healthy pregnant women at term with normal platelet count. After 24h of incubation, BeWo cells were lysed and subjected to qPCR analysis of *SERPINE1* expression (**C**) as well as to western blot analysis of PAI-1 (**D** and **E**). YWHAZ and GAPDH served as reference genes for gene expression analysis. Data are presented as mean ± SEM. BeWo cell data are from three independent experiments. $^{**}p \leq 0.01$, $^{****}p \leq 0.0001$

Supplemental Figure 2

CD39 and CD73 are upregulated in early onset preeclampsia

ENTPD1 (**A**) and NT5E (**B**) were analyzed from a previously published microarray dataset (Leavey et al., 2016) in preterm controls (n=25) and early onset preeclampsia (eoPE, n=25). Data are presented as mean ± SEM. $^{***}p \leq 0.001$, $^{*}p \leq 0.05$

Supplemental Figure 3

Anti-CD39 antibody comparison

Anit-CD39 antibodies from Invitrogen (clone JA90-36, left membrane) and Cell Signaling (clone E5A6L, right membrane) were compared using immunoblotting of first trimester and term placenta tissue lysates. Both blots had the same band pattern at approx. 70kDa, whereas anti-CD39 from Invitrogen showed some additional unspecific bands.

Supplemental Tables

Table S1

Primer Sequences

Gene	***Forward***	***Reverse***
**ENTPD1**	5'-GGA TGC GGG TTC TTC TCA CA-3'	5'-TCC AGG ACC TTT AAC CCT GC-3'
**NT5E**	5'-CTC CTC TCA ATC ATG CCG CT-3'	5'-TGG ATT CCA TTG TTG CGT TCA-3'
**YWHAZ**	5'-GGT GGC CAA TAT GGG GAT GT-3'	5'-TCC CTT TTA TTC CCC GCC AG-3'
**GAPDH**	5´-ACC CAC TCC TCC ACC TTT CA-3´	5´-CTG TTG CTG TAG CCA AAT TCG T-3´
**TBP**	5´-TGA CCC AGC ATC ACT GTT TC-3´	5´-CCA GCA CAC TCT TCT CAG CA-3´
**IL1B**	5'-TGA TGG CTT ATT ACA GTG GCA-3'	5'-GTG GTG GTC GGA GAT TCG TA-3'
**DDX3Y**	5'-AGT AGA GGC AAC CGG CAG TA-3'	5'-TGC ACT GGA GTA GGA CGA GTA-3'
**XIST**	5'-GAC ACA AGG CCA ACG ACC TA-3'	5'-TCG CTT GGG TCC TCT ATC CA-3'
**SERPINE1**	5'-GTT CTG CCC AAG TTC TCC CT-3'	5'-ACA TGT CGG TCA TTC CCA GG-3'

Table S2

List of Antibodies

Antibody	***Cat.#***	***Clone***	***Species***	***Distributor***	***Dilution IHC***	***Dilution WB***
**CD39**	91851	E5A6L	mAb rabbit	Cell Signaling		1:500
**CD39**	MA5-32707	JA90-36	mAb rabbit	Invitrogen	1:1000	1:500
**CD73**	13160	D7F9A	mAb rabbit	Cell Signaling	1:2000	1:1000
**PAI-1**	ab182973	EPR17272-21	mAb rabbit	abcam		1:1000
**β-actin**	Ab6276	AC15	mouse	abcam		1:500.000
**GAPDH**	2118	14C10	mAb rabbit	Cell Signaling		1:5000

Table S3. Baseline characteristics of the study groups

Table S3A

Study group for data shown in figure 1 – Gene expression

		**FT** (n=186)	**Term** (n=9)
Maternal age	years	26.34 (5.29)	35.29 (2.50)****
Maternal BMI (at delivery)	[kg/m^2]	21.52 (2.35)	28.52 (6.61)****
Maternal BMI (prepreg.)	[kg/m^2]	-	24.39 (5.37)
Gestational age	[days]	54.03 (11.90)	248.33 (18.46)****
Foetal weight	[g]	-	2592.00 (608.56)
Placental weight	[g]	-	496.67 (101.61)
Foetal sex	male/female	88/98	7/2
Smoker	yes/no	91/95	0/7
ThromboASS	yes/no	-	0/9

Data are presented as mean (±SD); FT vs. Term, ****p<0.0001

Table S3B

Study group for data shown in figure 1 – Protein levels

		**FT** (n=21)	**Term** (n=5)
Maternal age	years	28.95 (7.60)	34.75 (2.99)
Maternal BMI (at delivery)	[kg/m^2]	24.86 (3.50)	29.30 (6.91)
Maternal BMI (prepreg.)	[kg/m^2]	-	25.76 (5.29)
Gestational age	[days]	64.90 (15.03)	251.20 (15.29)****
Foetal weight	[g]	-	2608.40 (512.23)
Placental weight	[g]	-	560.00 (140.00)
Foetal sex	male/female	-	4/1
Smoker	yes/no	9/10	0/4
ThromboASS	yes/no	-	0/5

Data are presented as mean (±SD); FT vs. Term, ****p<0.0001

Table S3C

Study group for data shown in figure 3 – Gene and protein expression

		**Term** (n=12)	**PE** (n=12)
Maternal age	years	35.58 (5.58)	32.17 (6.73)
Maternal BMI (at delivery)	[kg/m^2]	27.62 (3.57)	30.80 (6.89)
Maternal BMI (prepreg.)	[kg/m^2]	23.65 (3.39)	25.77 (6.68)
Gestational age	[days]	269.08 (6.73)	239.08 (28.70)**
Foetal weight	[g]	3165.83 (429.70)	1865.09 (863.41)***
Placental weight	[g]	563.33 (128.72)	385.45 (155.59)**
Foetal sex	male/female	4/8	3/8
Smoker	yes/no	1/10	0/12
ThromboASS	yes/no	0/12	5/4

Data are presented as mean (±SD); Term vs. PE, **p ≤ 0.01, ***p ≤ 0.001

Table S3D
Study group for data shown in figure 4 – Gene and protein expression

		FT (n=3)	FT (n=6)	Term (n=5)
		gene expression	protein expression	gene and protein expression
Maternal age	years	26.33 (4.73)	26.67 (3.72)$^{\#}$	36.00 (5.83)
Maternal BMI (at delivery)	[kg/m2]	22.59 (3.60)	23.77 (4.45)	27.29 (3.10)
Maternal BMI (prepreg.)	[kg/m2]	-	-	21.68 (1.99)
Gestational age	[days]	52.33 (6.35)****	55.33 (5.43)$^{\#\#\#\#}$	269.40 (3.78)
Foetal weight	[g]	-	-	3350.00 (472.81)
Placental weight	[g]	-	-	628.00 (121.12)
Smoker	yes/no	3/0	4/2	0/5

Data are presented as mean (±SD); FT gene expression vs Term, ****$p \leq 0.0001$. FT protein expression vs. Term $^{\#}p \leq 0.05$, $^{\#\#\#\#}p \leq 0.0001$

Table S3E
Study group for data shown in figure 5 – Gene and protein expression

		FT (n=9)	FT (n=6)	Term (n=5)
		gene expression	protein expression	gene and protein expression
Maternal age	years	27.22 (5.78)*	26.67 (3.72)$^{\#}$	36.00 (5.83)
Maternal BMI (at delivery)	[kg/m^2]	21.78 (2.43)**	23.77 (4.45)	27.29 (3.10)
Maternal BMI (prepreg.)	[kg/m^2]	-	-	21.68 (1.99)
Gestational age	[days]	53.89 (5.51)****	55.33 (5.43)$^{\#\#\#\#}$	269.40 (3.78)
Foetal weight	[g]	-	-	3350.00 (472.81)
Placental weight	[g]	-	-	628.00 (121.12)
Smoker	yes/no	6/3	4/2	0/5

Data are presented as mean (±SD); FT gene expression vs Term, *$p \leq 0.05$, ****$p \leq 0.0001$. FT protein expression vs. Term $^{\#}p \leq 0.05$, $^{\#\#\#\#}p \leq 0.0001$

Table S4
Study group for data shown in supplementary figure 1

		Pre-term (n=25)	eoPE (n=25)
Maternal age	years	30.96 (5.65)	32.48 (6.34)
Maternal BMI	[kg/m^2]	24.29 (5.38)	28.94 (7.43)*
Gestational age	[days]	209.2 (19.04)	217.6 (13.25)
Foetal sex	male/female	16/9	10/15

Data are presented as mean (±SD); pre-term vs. early onset PE, *$p \leq 0.05$,

Supplemental methods

Measurement of platelet released PF4 and TGF-β

PF4 and TGF-β were measured in platelet releasates using commercial ELISAs for human PF4 (Human CXCL4/PF4 Quantikine, Cat#: DPF40; R&D Systems) and human TGF-beta 1 (Human TGF-beta1 Quantikine Kit, Cat#: DB100B; R&D Systems), according to the manufacturer's manuals.

Analysis of ENTPD1 and NT5E in previously published microarray datasets

The raw data and metadata for GSE75010 study was downloaded with the GEOquery R package (Davis and Meltzer, 2007). Background subtraction, quantile normalization, and summarization of raw microarray probe intensity values were done with RMA (Irizarry et al., 2003) function from the oligo R package. Custom CDF from Brainarray project (Sandberg and Larsson, 2007), version 25.0.0, was used to summarize and annotate the probes to Entrez Gene ID. Quality control was done with ArrayQualityMetrics (Kauffmann et al., 2009) and factoextra (http://www.sthda.com/english/rpkgs/factoextra) R packages. Differential expression analysis was performed with the limma R package (Ritchie et al., 2015). All the computations were done using Bioconductor version 3.16 (BiocManager 1.30) (Huber et al., 2015) and R version 4.2.2.

References to Supplemental material

Davis, S., and Meltzer, P. S. (2007). GEOquery: a bridge between the Gene Expression Omnibus (GEO) and BioConductor. *Bioinforma. Oxf. Engl.* 23, 1846–1847. doi: 10.1093/bioinformatics/btm254.

Huber, W., Carey, V. J., Gentleman, R., Anders, S., Carlson, M., Carvalho, B. S., et al. (2015). Orchestrating high-throughput genomic analysis with Bioconductor. *Nat. Methods* 12, 115–121. doi: 10.1038/nmeth.3252 [doi].

Irizarry, R. A., Hobbs, B., Collin, F., Beazer-Barclay, Y. D., Antonellis, K. J., Scherf, U., et al. (2003). Exploration, normalization, and summaries of high density oligonucleotide array probe level data. *Biostat. Oxf. Engl.* 4, 249–264. doi: 10.1093/biostatistics/4.2.249.

Kauffmann, A., Gentleman, R., and Huber, W. (2009). arrayQualityMetrics–a bioconductor package for quality assessment of microarray data. *Bioinforma. Oxf. Engl.* 25, 415–416. doi: 10.1093/bioinformatics/btn647 [doi].

Leavey, K., Benton, S. J., Grynspan, D., Kingdom, J. C., Bainbridge, S. A., and Cox, B. J. (2016). Unsupervised Placental Gene Expression Profiling Identifies Clinically Relevant Subclasses of Human Preeclampsia. *Hypertens. Dallas Tex 1979* 68, 137–147. doi: 10.1161/HYPERTENSIONAHA.116.07293.

Ritchie, M. E., Phipson, B., Wu, D., Hu, Y., Law, C. W., Shi, W., et al. (2015). limma powers differential expression analyses for RNA-sequencing and microarray studies. *Nucleic Acids Res.* 43, e47. doi: 10.1093/nar/gkv007 [doi].

Sandberg, R., and Larsson, O. (2007). Improved precision and accuracy for microarrays using updated probe set definitions. *BMC Bioinformatics* 8, 48–48. doi: 1471-2105-8-48 [pii].
